# 预充式CT引导下肺内小结节微弹簧圈穿刺定位法的初步探讨

**DOI:** 10.3779/j.issn.1009-3419.2019.06.04

**Published:** 2019-06-20

**Authors:** 凤卫 李, 应泰 陈, 建伟 边, 兴 辛, 迅 吴

**Affiliations:** 100076 北京，北京航天总医院胸外科 Department of Thoracic Surgery, Beijing Aerospace General Hospital, Beijing 100076, China

**Keywords:** 断层扫描, 定位, 肺肿瘤, Computed tomography, Localization, Lung neoplasms

## Abstract

**背景与目的:**

肺内小结节微弹簧圈穿刺定位是微创手术切除肺内小结节常用的术前定位方法，然而该方法仍有操作复杂、轻微并发症多等不足之处，我们将原有方法进行了优化。本研究旨在探讨优化后的预充式计算机断层扫描（computed tomography, CT）引导下肺内小结节微弹簧圈穿刺定位法在临床中的应用价值。

**方法:**

对2018年9月-2019年1月间31例患者的35枚肺结节，于术前采用预充式CT引导下肺内小结节微弹簧圈穿刺定位，然后施行胸腔镜下（video-assisted thoracoscopic surgery, VATS）手术。统计分析定位操作相关数据、成功率、并发症、病理结果等。

**结果:**

定位成功率97.1%，VATS切除成功率100%。CT定位时间平均10.1 min（5 min-31 min），微创切除病灶所需时间平均38.2 min（10 min-100 min）。术中发现微弹簧圈脱位回缩至胸壁内1例，通过充气膨肺状态下自胸壁穿刺点刺入穿刺针，成功定位肺内结节并予以切除。3例患者定位后发生微量气胸，但无需闭式引流处理。3例患者出现肺内血肿。35枚肺结节术后病理结果为：高分化腺癌15例，原位癌7例，微浸润腺癌5例，非典型腺瘤样增生4例，肺内淋巴结增生、炎性结节各2例。

**结论:**

采用预充式微弹簧圈定位肺内小结节简便、安全、有效，值得推广。

## Abstract (en)

**Background and objective:**

Preoperative computed tomography (CT) guided microcoil localization is a common method for small lung nodules before video-assisted thoracoscopic surgery (VATS). However, this method still has some limitation such as complicated operation and slight complications. We have optimized the original method. The purpose of this study was to investigate the clinical value of this optimized method.

**Methods:**

35 pulmonary nodules from 31 patients between September 2018 and January 2019 were localized by the optimized method before VATS. The success rate, complications, pathological results and localization operations related data were statistically analyzed.

**Results:**

The success rate of localization was 97.1%, and the success rate of VATS removal was 100%. The average operation time was 10.1 min (5 min-31 min), and the average time required for resection of lesions was 38.2 min (10 min-100 min). During the surgery, the microcoil of one patient was found to be dislocated and retracted into the chest wall. A puncture needle was inserted intolung tissue from the chest wall puncture point after the lung was inflated, and then the pulmonary nodule were successfully located and removed. A minor pneumothorax occurred in 3 patients, but no closed drainage was needed. Three patients developed intrapulmonary hematoma. The pathological results of 35 pulmonary nodules included 15 well-differentiated adenocarcinoma, 7 carcinoma *in situ*, 5 microinvasive adenocarcinoma, 4 atypical adenomatoid hyperplasia, 2 intrapulmonary lymph node hyperplasia, 2 inflammatory nodules.

**Conclusion:**

For small pulmonary nodules requiring thoracoscopic surgery, the optimized computed tomography-guided pulmonary nodule microcoil localization technique is convenient, safe and effective, and worthy of promotion to use.

随着大气污染和低剂量电子计算机断层扫描（computed tomography, CT）筛查的广泛应用，肺内小结节的发生率和检出率不断提高^[1]^。部分肺内小结节的病理为恶性，针对需要手术治疗的肺内小结节，首选的手术方式为胸腔镜手术^[2-5]^。肺内小结节胸腔镜术中定位困难，因此各种术前CT引导下定位技术应运而生^[6]^。其中微弹簧圈定位因严重并发症少、定位后可间隔较长时间再手术等优点，目前应用较为广泛，但其仍存在操作复杂、有一定比例的轻微并发症发生等问题^[7]^。为进一步优化操作流程、降低并发症，我们将原有方法进行了优化，采用预充式CT引导下肺内小结节微弹簧圈穿刺定位肺内小结节，取得较好临床效果，现将结果报道如下。

## 1 资料与方法

### 1.1 临床资料

#### 1.1.1 入选标准

（1）年龄在18岁（包含18岁）-85岁（包含85岁）之间；（2）临床可疑为恶性病变者；（3）无肺外远处转移者；（4）肺内病变需满足以下影像学标准：①纯磨玻璃影或实性成分≤25%的非纯磨玻璃影；②直径≤1 cm的实性结节或实性成分≤1 cm的非纯磨玻璃影，且结节的实性成分与脏层胸膜距离≥0.5 cm；（以上两条需满足一条）；③无胸膜牵拉征或累及胸膜。

#### 1.1.2 排除标准

（1）病灶部位不适合经皮肺穿刺定位者；（2）合并气胸、胸腔积液者；（3）一般情况差，心肺功能严重损害、恶病质，不能耐受手术者；（4）拒绝手术治疗者；（5）未签署知情同意书者。

经北京航天总医院伦理委员会批准，并在美国临床试验注册中心成功注册（注册号：NCT03649906）。筛选2018年9月-2019年1月在北京航天总医院胸外科进行胸腔镜手术的肺内小结节患者121例，其中因病变较小预计术中无法定位需要术前行微弹簧圈定位者33例，除外2例拒绝入组者，最终入组31例患者包含35枚结节，其中28例为单发结节，2例2枚结节，1例3枚结节，患者一般情况见表 1。

**1:** 患者一般情况 Clinical characteristics of 31 patients

Variables	*N* =31 (patients)	
Age (Mean±SD, yr)	58.1±13.0	
Gender		
Male	12（38.7%）	
Female	19（61.3%）	
Location of nodules	35	
Right upper lobe	6	
Right middle lobe	7	
Right lower lobe	5	
Left upper lobe	10	
Left lower lobe	7	
Diameter of nodules (Mean±SD, mm) Density of nodules	8.6±4.1	
Pure GGO Mixed GGO Solid nodules	25（71.4%） 5（14.3%） 5（14.3%）	
Distance from nodule to pleura [median (Q1, Q3), mm]	14.0（9.5, 28.8）	
GGO: ground-glass opacity.	

### 1.2 设备与材料

CT机（美国GE Lightspeed 16排螺旋CT），CT定位标尺，穿刺针（22 G，美国Angiotech），栓塞微弹簧圈（COOKMicrocoil金属丝直径0.018' ' 长度9 cm），10 mL注射器，2%利多卡因。

### 1.3 穿刺方法

首先将微弹簧圈充填入22 G穿刺针针体，针尾部以透明贴膜密封，备用；根据影像资料结合手术方案，选择所需的体位，粘贴CT定位标尺，进行扫描，选择最佳穿刺点，测量进针深度、进针角度，常规消毒铺巾，2%利多卡因局麻胸壁软组织，将预充有微弹簧圈的22 G穿刺针按照所测量角度和深度刺入胸壁软组织，注意不能刺破壁层胸膜，重复CT扫描，如穿刺针的位置和角度满意，则将穿刺针针尖刺破胸膜推至目标位置；如CT扫描发现进针点、进针角度存在偏差，则予以调整，直至穿刺针位置、角度合适后，再进针将穿刺针针尖刺破胸膜推至目标位置。重复CT扫描，确认针尖位于目标位置后，以22 G穿刺针针芯刺破以透明贴膜密封之预充有微弹簧圈的22 G穿刺针针体，分别先于病灶周围释放部分微弹簧圈，缓慢退针至壁层胸膜下再次释放剩余部分微弹簧圈。完成操作后再次重复CT扫描确认效果，完成定位，见图 1。

**1:**
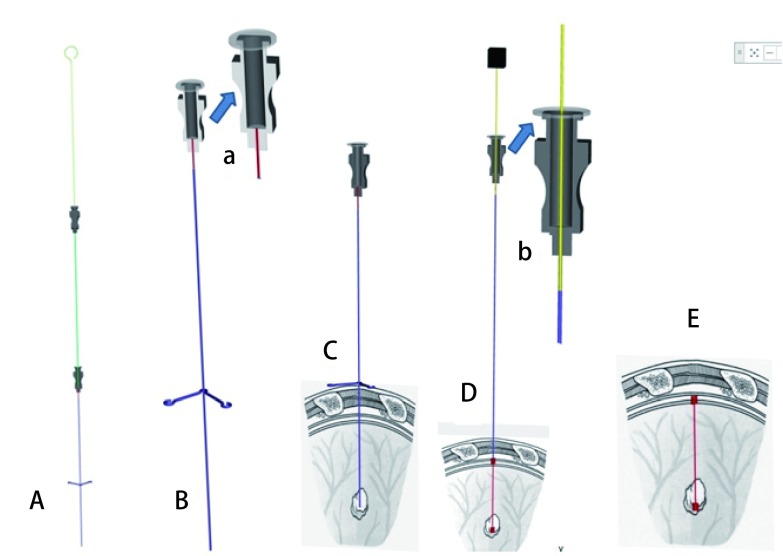
预充式微弹簧圈穿刺法操作流程：A：首先将弹簧圈推送入穿刺针针道内；B：以薄膜封堵穿刺针针尾，防止漏气（a.以透明薄膜封堵穿刺针针尾, 施乐辉IV3000 7 cm×9 cm）；C：将预装有弹簧圈并且针尾以薄膜封堵了的穿刺针刺入肿物旁）；D：以穿刺针针芯刺破薄膜并推送释放弹簧圈(b.先将穿刺针针芯刺破薄膜进入针道以释放部分微弹簧圈，然后稳定针芯缓慢退穿刺针至壁层胸膜外，再次推进针芯完全释放剩余微弹簧圈)；E：弹簧圈释放完毕，分别盘旋于肺表面及肿物旁。上述流程中穿刺针针道与大气完全隔绝，减少了气胸的发生。 Operation procedure of optimized CT-guided pulmonary nodule microcoil localization technique. A: Prefill the microcoil into the puncture needle; B: Seal the tail end of the puncture needle (a. seal the needle tail with a transparent film, IV3000 7 cm×9 cm Smith & Nephew); C: Puncture the pre-filled needle into the tumor; D: Release the microcoil (b. Puncture the film with the needle core and into the needle to release a part of the microcoil, then hold the needle core and withdraw needle to pleura, and then push the rest needle core to release all of the microcoil); E: The microcoil is released, hovered over the surface of the lungs and next to the tumor; In the above process, the needle cavity is completely isolated from the atmosphere, which theoretically reduces the occurrence of pneumothorax.

### 1.4 手术方法

胸腔镜下全面探查胸腔，通过操作口进行触诊，结合定位影像确定结节的位置，必要时行术中X线协助，并以电凝钩烧灼标记病灶，先以卵圆钳夹持微弹簧圈、结节以及周围少量正常肺组织，用内镜直切割缝合器楔形切除病灶，或行解剖性肺段/肺叶切除，送术中快速病理。根据快速病理结果决定下一步手术方案。

## 2 结果

### 2.1 定位相关结果

2018年9月-2019年1月间，采用预充式微弹簧圈定位肺内小结节患者31例共35枚结节，平均定位时间10.1 min，34枚结节定位成功（97.1%），术中发现微弹簧圈脱位回缩至胸壁内1例，通过充气膨肺状态下自胸壁穿刺点刺入穿刺针，成功定位肺内结节并予以切除。3例患者定位后发生微量气胸，但无需闭式引流处理。3例患者出现肺内血肿，其中1例血肿较明显，原因为操作中微弹簧圈意外盘旋于穿刺针针尾，故该穿刺针针道无微弹簧圈填充，出现约4 cm×3 cm范围血肿，予重新穿刺并成功定位后胸腔镜下切除病灶，其余2例血肿均为CT下显示针道周围少量斑片影，术中胸腔镜下无异常表现。多数患者大约于操作后1 h-2 h开始出现穿刺处轻微酸痛，2例（6.5%）患者因疼痛较剧烈需镇痛药物治疗。患者定位情况见表 2。

**2:** 患者定位相关情况 Procedural related parameters

Variables	*N*=31 (patients)
Time interval from localitation to surgery	
On the day of surgery	1（3.2%）
The day before of surgery	30（96.8%）
Number of CT scans (Mean±SD)	4.6±1.0
Average procedural time (Mean±SD, min)	10.1±5.0
Success rate	34/35（97.1%）
Complication	
Pneumothorax	3（9.7%）
Hematomas	3（9.7%）
Chest pain	2（6.5%）
Hemoptysis	0
Gas embolism	0

### 2.2 手术相关结果

胸腔镜下手术31例患者35枚结节，无中转开胸者。术式包括楔形切除17例、肺段切除9例、楔形切除后行肺叶切除6例、直接肺叶切除3例。术后病理显示恶性结节27枚，包括肺腺癌15例，原位癌7例，微浸润腺癌5例。非典型腺瘤样增生4枚，肺内淋巴结增生、炎性结节各2枚。具体见表 3。

**3:** 手术方式及术后病理情况 Surgical and pathological parameters

Variables	*N*=35 (nodules)
Surgical methods	
Wedge resection	17（48.6%）
Segmentectomy	9（25.7%）
Wedge resection+Lobectomy	6（17.1%）
Lobectomy	3（8.6%）
Postoperative pathology	
Invasive carcinoma	15（42.9%）
Minimally invasive adenocarcinoma (MIA)	5（14.3%）
Adenocarcinoma in situ (AIS)	7（20%）
Atypical adenomatoid hyperplasia (AAH)	4（11.4%）
Intrapulmonary lymph node hyperplasia	2（5.7%）
Inflammatory nodules	2（5.7%）

## 3 讨论

电视胸腔镜手术在肺内小结节的手术治疗中发挥着主导作用，而肺内小结节因其体积小、质地软，与正常肺组织区分度低，胸腔镜手术中术者的手无法深入胸腔内进行完整触摸探查，因而术中触诊定位成功率低，触诊失败率为63%-88%^[8, 9]^，常因无法定位肺内小结节而中转开胸手术，甚至直接行病变所在肺叶切除术，明显增加了患者创伤、影响术后恢复。为帮助胸腔镜手术中寻找肺内小结节，各种肺内小结节术前定位方法应运而生，具体方法为手术前在CT的引导下于病变周围放置各种标志物，包括金属材料的带钩钢丝、螺旋金属丝、微弹簧圈；液体材料的美兰、碘油、稀硫酸钡、放射性核素等，上述方法各有优缺点。美兰等液体染料定位存在染料扩散或消失、肺组织碳末沉积导致色素沉着的患者不易寻找定位点、肺结节被染料染色后对病理结果的判读带来一定困难等问题，目前临床应用较少^[10]^。目前应用较为广泛的包括微弹簧圈和带钩钢丝。带钩钢丝简单易行，定位成功率高。但肺组织疏松且充气，带钩钢丝易发生脱落，容易产生气胸，所以往往需要定位后短时间内手术，从而限制了该方法的应用^[11]^。微弹簧圈因结构纤细、质地柔软、包裹有促凝作用的纤维丝，可及时有效地堵塞穿刺针眼，故而有气胸发生率相对低的优点，我们曾报道1例微弹簧圈定位后因术前突发心梗而推迟3月再行手术者，期间未发生任何并发症，故微弹簧圈定位后可从容安排手术^[12]^。

大部分文献报道采用微弹簧圈定位的操作时间为23 min-72 min^[13, 14]^。随着人群健康意识的提高以及医保覆盖范围的提高，放射科工作量常常处于饱和状态，CT引导下穿刺定位需要占用放射科相对长的时间，而定位收费却是按照次数收费，因此导致胸外科和放射科沟通不畅，甚至无法满足全部患者的定位需要，定位操作时间越长这种矛盾就越明显。为解决这一问题，我们发现穿刺过程中穿刺针位置改变，进而出现微弹簧圈嵌顿、残留，以及气胸等并发症的发生是造成定位操作时间长的主要原因。基于这一发现，我们开始设想并尝试了一系列技术优化措施，最终锁定将微弹簧圈预充填于穿刺针内并密封再进行后续穿刺的预充式微弹簧圈肺内小结节穿刺定位法。该方法将充填步骤穿插于扫描操作的间隙，操作更简便，用时更短，实际应用过程中我们的操作用时为10.1 min，短于文献所报道时间，也支持这一假设。

气胸为肺穿刺定位的最常见并发症，定位操作中一旦发生气胸可引起患者胸闷、憋气症状，增加脱位的可能性^[15]^。与国内外文献研究报道的13.2%-33%的气胸发生率不同^[7, 16-18]^，本组病例气胸发生率为9.7%。为降低气胸发生率，除了应用我们之前的经验外^[12]^，尽可能减少穿刺针针道与大气相通的时间亦为有效的办法。首次报道微弹簧圈穿刺定位法的Powell等使用导丝将微弹簧圈装载入22 G穿刺针后未密封穿刺针即直接穿刺，穿刺针针道全程与大气相通无疑增加了气胸风险^[19]^。国内较早应用微弹簧圈穿刺定位的杨峰等为改进这一问题采取先穿刺病灶，位置满意后再进行装载和释放弹簧圈，有效减少了穿刺针针道与大气相通的时间，但将加载微弹簧圈的过程由穿刺针的准备阶段转移到了人体穿刺阶段增加了操作难度^[20]^。本组患者我们采用将微弹簧圈预充填于穿刺针内并密封再进行后续穿刺的预充式微弹簧圈肺内小结节穿刺定位法。该方法避免了操作过程中穿刺针道与大气相通，缩短了操作时间，在一定程度上进一步降低了气胸的发生率。当然较低的气胸发生率与我们入组病例少可能也有关系，需要进一步扩大样本量验证该结果。

本组病例定位成功率为97.1%。本组有1例患者CT显示定位成功，胸腔镜手术时发现微弹簧圈脱位回缩至胸壁，分析原因为定位该结节时微弹簧圈留置于肩胛骨活动范围内，定位成功后肩胛骨的活动导致了微弹簧圈的脱位。我们在后续的操作中对于肩胛骨区域的结节定位时，进针点尽量避开肩胛骨活动范围或定位成功后嘱患者患侧上肢固定。既往文献报道^[20, 21]^定位失败的主要原因为微弹簧圈脱位，微弹簧圈脱位高危因素为穿刺路径经过叶间裂和穿刺针刺入肺实质过浅。本组患者操作过程中穿刺针刺入肺实质内的深度均大于1 cm，并且避免了穿刺路径距离叶间裂过近，在一定程度上降低了脱位的发生。同时我们较高的成功率可能与本组病例数较少以及新方法开展早期操作人员的谨慎态度和病例选择有关。

本组出现较明显的肺内血肿1例，原因为该患者预充填微弹簧圈时误充填于穿刺针针尾处，未能及时发现而按原步骤进行后续操作，直至CT扫描确定微弹簧圈位置时发现肺内出现血肿，且病灶周围并无微弹簧圈，通过检查发现微弹簧圈藏匿于穿刺针针尾，所幸该患者仅出现血肿并无气胸发生，重新装填后完成肺结节定位。在此后的病例中我们增加了预充填微弹簧圈后检查针尾的步骤，避免了此类情况的再次出现。多数需要术前定位的病灶是直径小、质地软难以术中定位的小结节，此类病灶如位于外周多可先行局部切除根据术中快速病理决定具体手术方式，本组有3例患者定位后直接行肺叶切除，其中1例因病灶多发，1例因病灶较深邻近肺门，1例因肺内大病灶所在肺叶内合并存在1枚结节，故而术前定位在切除肺叶后寻找全部病灶、缩短寻找病灶的时间方面亦具有一定价值。

相比传统的微弹簧圈定位法，操作中我们使用穿刺针配套的针芯替代导丝推出微弹簧圈，完成释放过程，节省了约800元/患者，减轻了患者的经济负担。然而本操作方法在穿刺前将微弹簧圈预充填于穿刺针内，如果患者因局麻药过敏、无法耐受穿刺体位、结节消失等原因临时取消操作势必会带来材料上的浪费。此外，我们曾经有1例患者因胸壁较厚，普通10 mL注射器所附带针头无法完全局麻浸润，遂改用22 G穿刺针局麻，局麻后发现该穿刺针无法装填微弹簧圈，分析原因为针道内有局麻药残留，浸湿了微弹簧圈的促凝纤维，增大了微弹簧圈与针道的阻力所致，因此建议如果需要用穿刺针进行局麻的，应更换新穿刺针进行装填微弹簧圈。

综上，采用预充式微弹簧圈定位肺内小结节简便、安全、有效，值得推广。
